# A Randomized, Double-Blind, Placebo-Controlled Trial to Assess the Effectiveness and Safety of Melatonin and Three Formulations of Floraworks Proprietary TruCBN™ for Improving Sleep

**DOI:** 10.3390/ph17080977

**Published:** 2024-07-24

**Authors:** Antonija Kolobaric, Jessica Saleska, Susan J. Hewlings, Corey Bryant, Christopher S. Colwell, Christopher R. D’Adamo, Jeff Chen, Emily K. Pauli

**Affiliations:** 1 Radicle Science, Inc., Del Mar, CA 92014, USAjessica@radiclescience.com (J.S.); corey.bryant@radiclescience.com (C.B.);; 2 Center for Neuroscience, University of Pittsburgh, Pittsburgh, PA 15260, USA; 3 Department of Psychiatry & Biobehavioral Sciences, University of California–Los Angeles, Los Angeles, CA 90095, USA; 4 Department of Family and Community Medicine, University of Maryland School of Medicine, Baltimore, MD 21201, USA

**Keywords:** cannabinol (CBN), phytocannabinoids, sleep, PROMIS, decentralized clinical trial

## Abstract

The phytocannabinoid cannabinol (CBN) has a potential mechanism of action as an alternative sleep aid but there is minimal evidence to support its effectiveness. The aim of this randomized, double-blind, placebo-controlled study was to assess the safety and effects of three formulations of a hemp-derived CBN sleep aid, TruCBN™ [25 mg (n = 206), 50 mg (n = 205), 100 mg (n = 203)], on sleep quality (PROMIS Sleep Disturbance 8A), relative to placebo (n = 204). The effectiveness and safety of these formulations relative to 4 mg of melatonin (n = 202) was assessed. Exploratory measures were stress (PROMIS Stress 4A), anxiety (Anxiety 4A), pain (PROMIS™ PEG), and well-being (WHO 5). All groups and the 4 mg melatonin group experienced significant improvement in sleep quality relative to the placebo group with no significant differences between any group and the melatonin group. Participants taking 100 mg showed a larger decrease in stress compared to the placebo group. There were no significant differences in anxiety, pain, well-being, or the frequency of side effects between any group and the placebo group. There was no significant difference in improvements in sleep quality between any of the treatment groups and the 4 mg melatonin group. Orally ingested CBN, at 25 mg, 50 mg, and 100 mg, is a safe and effective alternative for the improvement of sleep.

## 1. Introduction

Sleep deprivation can have a profound impact on overall well-being, negatively impacting brain function [1], cognitive performance [2], emotional well-being [2,3], and physical health [4,5]. Inadequate sleep is associated with poor overall mental health as well as increased perceived stress and anxiety [6]. Individuals who are sleep deprived are less productive and report a lower overall quality of life [7]. Despite these consequences, about one in three American adults do not get sufficient sleep each night [8]. Robust clinical evidence supports the use of prescription drug interventions, such as benzodiazepine receptor agonist drugs, for treating sleep disorders [9,10]. Nevertheless, concerns persist as to whether they are effective in the long term, and over their numerous side effects, including the considerable risk of abuse and dependence [11]. Safe and accessible alternative therapies must be evaluated to improve the well-being of those suffering from sleep difficulties. Melatonin is a popular alternative to prescription sleeping medications with a large amount of clinical data to support its efficacy. Because of its widespread use and clinical effectiveness, melatonin is often the sleep aid to which other non-prescription interventions are compared [12,13,14].

Cannabis preparations have also gained increasing attention as potential alternative therapies for addressing sleep disturbances [15]. Cannabinoids exert diverse effects on the human body through their interaction with the endocannabinoid system (ECS), which is distributed throughout the brain, central nervous system, and peripheral nervous system [16]. The presence of the ECS in the hypothalamic–pituitary–adrenal axis (HPA) and sympathetic nervous system support its role in regulating stress, feelings of anxiety, and pain [17]. Additionally, the ECS has been proposed to regulate the circadian sleep/wake cycle, suggesting that cannabinoids play a role in modulating sleep and potentially aspects of health that sleep impacts such as stress and anxiety [18]. The effect of the common phytocannabinoids, delta-9 tetrahydrocannabinol (THC), and cannabidiol (CBD), on sleep has been studied and is well supported by the role of the ECS on circadian regulation [19].

Cannabinol (CBN), a minor phytocannabinoid, has rapidly grown in popularity as a sleep aid, with many manufacturers claiming that it has sleep-inducing effects [20].

Yet, despite anecdotal evidence and a plausible mechanism of action via the ECS, there is limited research on the compound’s effect on sleep. Preclinical and anecdotal evidence suggests that CBN could prolong sleep [21], though to our knowledge no published blinded randomized placebo-controlled trials have studied CBN’s effectiveness for disturbed sleep without combining it with other ingredients. Rigorous large-scale clinical trials are needed to assess the dose, efficacy, and safety of CBN for sleep.

The primary aim of the study was to assess the safety and effects of three formulations of softgels containing varying amounts of TruCBN™ (25–50–100 mg) on sleep quality, relative to a placebo control. As a secondary aim, we also sought to assess the comparative effectiveness and safety of these CBN products relative to a softgel containing 4 mg of melatonin. Furthermore, because of the interconnection between sleep, stress, anxiety, pain, and overall well-being, and the potential effect that CBN may have on these indices because of its effect of the ECS, we sought to include the effect on these outcomes as exploratory measures. 

## 2. Results

### 2.1. Participants

The study included participants with a sex distribution of 54% female and 46% male, while 80% identified their race as white. After stratification, the participant numbers in each group were as follows: softgel A (202), softgel B (206), softgel C (205), softgel D (203), and placebo (204). There were no significant differences observed between the groups in terms of demographic or outcome variables at baseline (Table 1).

### 2.2. Sleep

**Figure 1:**
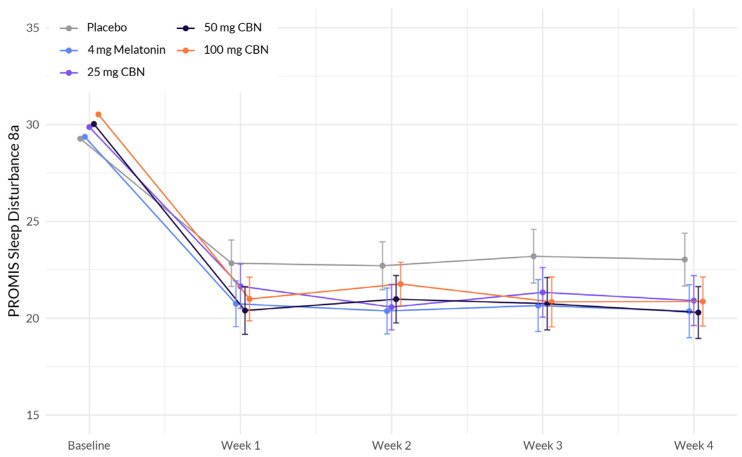
Comparison of mean PROMIS Sleep Disturbance 8a score change between active arms and placebo in adults with sleep disturbance.

The interaction between study week (there are six assessment time points referred to as study weeks) and softgel A (melatonin), showed a significant negative association with change in sleep disturbance (β = −0.564, *p* = 0.029) (Figure 1 and Table 2). This indicates that the effect of study week on sleep disturbance differed between the treatment groups, with participants in the softgel A group experiencing a greater reduction in sleep disturbance over time compared to the placebo group. The same pattern was observed for the other treatment groups: softgel B (β = −0.544, *p* = 0.030), softgel C (β = −0.603, *p* = 0.018), and softgel D (β = −0.566, *p* = 0.023). Next, we computed a post hoc analysis to compare the difference between active arms containing TruCBN™ and melatonin (softgel A). Contrast analyses underwent Bonferroni correction and revealed no statistically significant differences in effects between softgel A and softgel B (t = 0.32, *p* > 0.05), softgel A and softgel C (t = −0.09, *p* > 0.05), or softgel A and softgel D (t = 0.06, *p* > 0.05). Finally, we did not observe any significant differences in achieving a minimum clinically important difference (MCID) between softgel A (estimate = 1.43, 95% CI [0.7, 2.16], *p* = 0.434), softgel B (estimate = 1.33, 95% CI [0.65, 2.01], *p* = 0.729), softgel C (estimate = 1.46, 95% CI [0.72, 2.2], *p* = 0.348), or softgel D (estimate = 1.39, 95% CI [0.7, 2.08, *p* = 0.498]) and placebo (42.2%). (MCID is defined as a change in one half the standard deviation of the baseline). This means there was no difference in the MCID achieved in the placebo group and that of any of the active groups.

### 2.3. Anxiety

**Figure 2:**
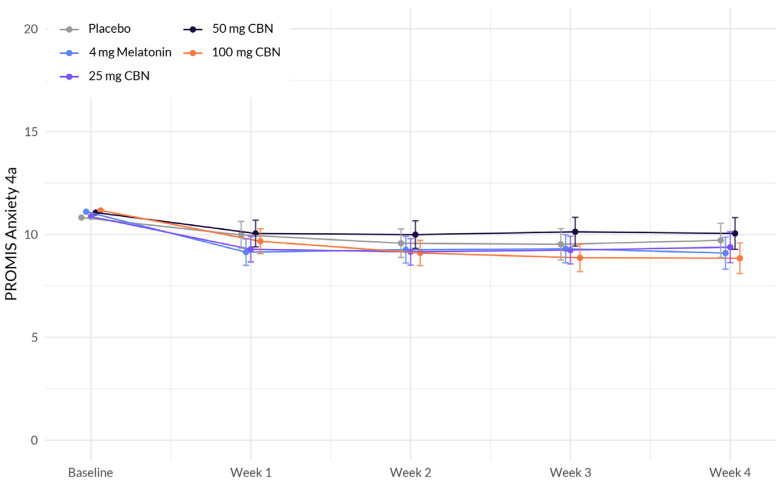
Comparison of mean PROMIS Anxiety 4a score change between each active arm and placebo in adults with sleep disturbance. There was no significant difference between actives and placebo.

The analysis revealed several associations with anxiety (Figure 2 and Table 3). Education demonstrated a significant positive association with change in anxiety (β = 1.141, *p* < 0.001), indicating that individuals without a college degree reported higher levels of anxiety. BMI also exhibited a significant positive association with anxiety (β = 0.032, *p* = 0.007), suggesting that a higher BMI was associated with higher levels of anxiety. However, the interactions between study week and softgel A (Arm 1) (β = −0.095, *p* = 0.490), softgel B (β = −0.028, *p* = 0.838), softgel C (β = 0.130, *p* = 0.346), and softgel D (β = −0.232, *p* = 0.086) did not reach statistical significance at the 0.05 level. Therefore, the effect of study week on anxiety did not significantly differ between the treatment groups compared to the placebo group.

### 2.4. Stress

**Figure 3:**
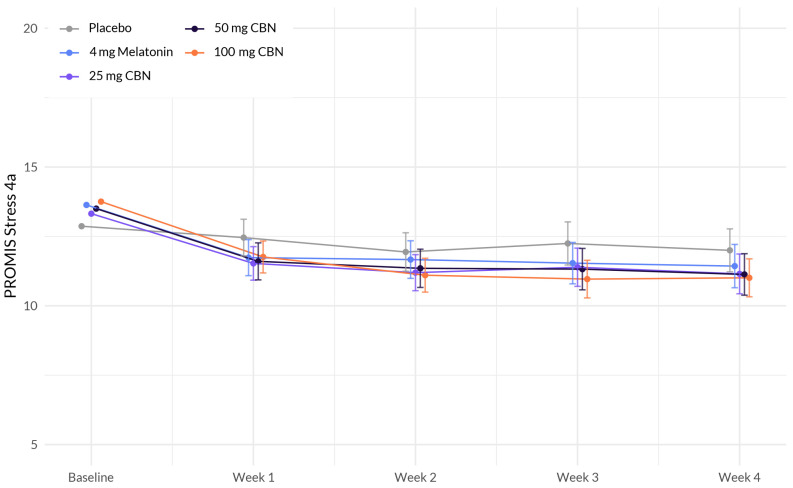
Comparison of mean PROMIS Stress 4a score change between each active arm and placebo in adults with sleep disturbance. 100 mg CBN led to a significant larger decrease in stress compared to placebo.

The analysis revealed several associations with change in stress (Figure 3 and Table 4). While there was no significant interaction between study week and softgel A (Arm 1) (β = −0.189, *p* = 0.162), softgel B (β = −0.195, *p* = 0.132), and softgel C (β = −0.228, *p* = 0.085), a significant negative interaction was observed between study week and softgel D (β = −0.323, *p* = 0.011). This indicates that the effect of study week on stress levels differed between the treatment groups, with participants in softgel D showing a larger decrease in stress over time compared to the placebo group.

### 2.5. Pain

We did not detect any significant interactions between study week and softgel A (Arm 1) (β = −0.081, *p* = 0.401), softgel B (β = −0.125, *p* = 0.1680), softgel C (β = 0.057, *p* = 0.5491), and softgel D (β = −0.119, *p* = 0.1888) at the 0.05 level. Therefore, the effect of study week on pain did not significantly differ between the treatment groups compared to the placebo group. Several demographic variables were significantly associated with change in pain.

### 2.6. Overall Well-Being

We did not detect any significant interactions between study week and softgel A (Arm 1) (β = 0.302, *p* = 0.079), softgel B (β = 0.280, *p* = 0.093), softgel C (β = 0.194, *p* = 0.252), and softgel D (β = 0.243, *p* = 0.143) at the 0.05 level. Therefore, the effect of study week on change in overall well-being did not significantly differ between the treatment groups compared to the placebo group.

### 2.7. Side Effects

**Figure 4:**
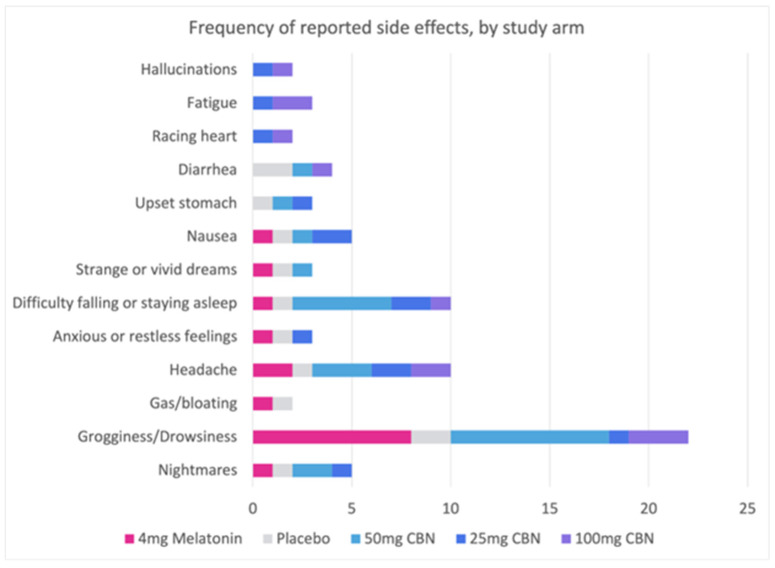
Comparison of side effects between active and placebo groups. This figure displays the occurrence of side effects in the active and placebo groups. All side effects were mild and non-serious, requiring no emergency or non-emergency healthcare services.

There was no significant difference in the frequency of reported side effects between the placebo group and the other groups (χ^2^ (4) = 8.58, *p* = 0.073), as shown in Figure 4. Participants in the study reported a range of side effects, including grogginess/drowsiness (n = 22), difficulty falling or staying asleep (n = 10), headache (n = 10), nausea (n = 5), nightmares (n = 5), diarrhea (n = 4), upset stomach (n = 3), strange or vivid dreams (n = 3), anxious or restless feelings (n = 3), hallucinations (n = 2), racing heart (n = 2), and gas/bloating (n = 2). Additionally, each of the following side effects was reported by one participant each: chest pain, constipation, urinary tract infection, tingling in lips, dry mouth, weight gain, dry or itchy eyes, low libido, rash, and brain fog. Side effects were mild; none were considered serious or required the use of emergency or non-emergency healthcare services.

A Summary of Key Findings

- We observed a significant difference in effect on sleep between 50 mg of CBN and placebo and between 4 mg of melatonin and placebo.
- We observed a marginally significant difference in effect on sleep between 25 mg of CBN and placebo and between 100 mg of CBN and placebo.
- No significant differences in effect on any other health outcomes were observed between the active product arms and placebo control.
- All side effects were mild or moderate. There were no significant differences in the frequency of reported side effects between the active and placebo arms.

## 3. Discussion

The primary analyses of this study revealed significant improvements in sleep outcomes as measured by the PROMIS Sleep Disturbance 8a score. The key findings were a significant difference in the rate of mean PROMIS Sleep Disturbance 8a score change between all groups relative to the placebo group, indicating that the 25, 50, and 100 mg serving sizes of TruCBN™ were effective in improving sleep disturbances. Additionally, the group taking 4 mg of melatonin reported significant improvements in sleep outcomes compared to the placebo group. All side effects were mild or moderate. There were no significant differences in the frequency of reported side effects between any dose of TruCBN™ formulations or melatonin compared to placebo.

While CBN is a popular alternative to prescription drugs to improve sleep outcomes, there are few well-designed clinical trials to assess its effectiveness, especially as a single ingredient. The findings of this study are consistent with previous findings, according to which a combination of cannabinoids could improve sleep. For example, a randomized, controlled crossover trial administering a combination product containing THC 20 mg/mL, CBN 2 mg/mL, CBD 1 mg/mL, and naturally occurring terpenes (extracted from the cannabis plant) in pharmaceutical grade sunflower oil for 2 weeks demonstrated an improvement in sleep quality in subjects with insomnia when compared to placebo [22]. In a recent study, we reported that a botanical blend containing a low concentration of THC, CBD, and CBN improved sleep disturbance, anxiety, stress, and well-being in healthy individuals who reported better sleep as a primary health concern [23]. In a similar study, subjects receiving a tablet containing 10 mg THC and 5 mg CBN nightly experienced significantly improved sleep quality and slept significantly longer, with a 5% increase in sleep duration [20]. We recently published the results of a similar sleep study on 1793 adults [24]. Participants were randomly assigned to take 1 of 6 products containing either 15 mg CBD or 5 mg melatonin, alone or in combination with minor cannabinoids, including CBN. Most participants (56% to 75%) across all formulations experienced a clinically important improvement in their sleep quality, though not statistically better than the active control group that took 5 mg of melatonin alone [24]. Our results are further supported by the role CBN plays on the ECS. However, to our knowledge, this is the first study of this size to demonstrate the effectiveness of highly purified CBN isolate formulations containing 25, 50, and 100 mg doses of TruCBN™ for improvement in sleep outcomes.

This study intended to assess the “real-world” effectiveness of varying doses of CBN by administering them to a broad population representative of a consumer seeking such a product for sleep disturbances and the other outcomes studied. This is in contrast to traditional clinical trials, which often have restrictive eligibility criteria, rigorous monitoring, and are limited to those who can access the site being utilized to conduct the trial. As a result, traditional trials often exhibit higher levels of missingness and heterogeneity and lack external validity, as the participants’ characteristics and behaviors may not accurately represent those of real-world users. It was the goal of this study to reflect the real-world effects of the study products and to offer a distinct and unique way to provide evidence for regulatory and clinical decision-making and additional clinical trial design [25].

The study had a few limitations. First, approximately 26% of participants did not complete any follow-up surveys. However, the overall attrition level was still below our anticipated attrition (45%) and the study remained adequately powered to detect significant sleep changes. We were also unable to perform a sensitivity analysis including excluded participants, as they did not provide any PROMIS Sleep Disturbance 8a scores. Thus, including their data would not be appropriate as their data were determined to be non-randomly missing. Nevertheless, there were no significant differences in baseline demographic or health characteristics between study arms in the final study sample. This indicates that balance was maintained across study arms. In addition, a covariate regression analysis was performed to further account for potential sources of confounding.

This study supports the safety of CBN with mild to moderate side effects reported over six weeks. This is similar to other studies administering CBN to healthy subjects [23,24]. 

## 4. Materials and Methods

This study, Radicle^TM^ Rest, was a randomized, double-blind, placebo-controlled parallel trial designed to assess the effects of 3 formulations of TruCBN™ and 1 formulation of melatonin softgels on sleep, anxiety, stress, pain, and overall health-related quality of life. The study was decentralized; participants did not attend any in-person visits and all data were collected via online surveys which participants accessed via participant specific hyperlinks sent to them at scheduled times through their preferred means of communication (email or SMS text). Participants were recruited online from across the United States through social media, Radicle Science’s electronic mailing list, and a third-party consumer network with nationwide representation. Recruitment emails containing links to the study screener were sent to those within the Radicle Science mailing list and consumer network, while social media advertisements led to a study landing page with a link to the study screener. Participants were eligible if they were 21 years old or older, resided in the United States, expressed a desire for better sleep, and ranked their desire for better sleep as a primary reason for taking a dietary supplement. Individuals were excluded if they were pregnant or breastfeeding, or taking medications that posed a health risk when used in conjunction with any of the study product ingredients. Eligible individuals were directed to a secure online portal to provide informed consent. Participants indicated their consent electronically by signing the informed consent form and were sent a digital copy of the electronic consent. Eligible individuals were advised to consult with their healthcare provider before participating if they had a diagnosed medical condition, were on any prescription medication or supplements, or had any upcoming medical procedures planned. Immediately following informed consent, participants completed an intake survey which collected basic demographic information, health behaviors, and experienced sleep quality. This research process has been successfully implemented for several other dietary supplement clinical trials [23,24].

**Figure 5:**
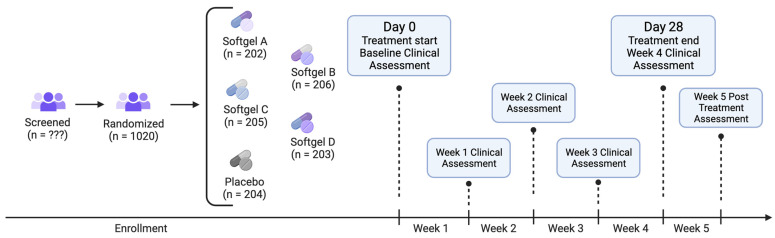
Study flow diagram.

Recruits who consented to participate and completed intake were randomized to one of five study arms (see below for details on randomization): (1) softgel A (containing 4 mg melatonin), (2) softgel B (containing 25 mg CBN (TruCBN™), (3) softgel C (containing 50 mg CBN), (4) softgel D (containing 100 mg CBN) and (5) placebo control. All study products were provided by the partnering manufacturer. After receiving all study products at our warehouse, and before shipping them to participants, we sent samples from each arm to an independent laboratory to ensure active ingredient presence, potency, and lack of contaminants. Participants were instructed to take one softgel 1–2 h before bedtime. The study was double-blind; neither the participants nor those who collected and analyzed the data were aware of the product that participants received until the conclusion of the study. Upon receiving the product, participants were asked to verify the study product alpha-numeric identification coder on their product to ensure that they received their assigned product. For 5 weeks following the study initiation and baseline health outcome assessment (4 weeks after taking the study product and 1 week after finishing the study product), participants were asked to complete online surveys, which they accessed via unique hyperlinks sent at scheduled times via email or text (see Figure 5 for Study flow). During the baseline week, participants completed health outcome assessments of their sleep, feelings of anxiety, stress, pain, and overall well-being, using validated, patient-reported outcome measures.

Throughout the study duration, participants electronically received a health survey asking them to report their study product usage and health outcome assessments for their sleep disturbance, feelings of anxiety, stress, pain, and overall well-being from the past week using the same validated health measures used at baseline (Table 5). In every study survey, following receipt of their product, participants were also prompted to report any side effects and were encouraged to contact the research team directly if they experienced side effects at any point.

The Sterling Institutional Review Board (SIRB) approved the study [10147-EKPauli]. The study was registered on ClinicalTrials.gov [NCT05511818] on 21 August 2023.

### 4.1. Randomization

Participants were randomly assigned to one of the five study product arms, with an equal chance of being assigned to each group (1:1:1:1:1 ratio). Prior to randomization, participants were stratified by their assigned sex at birth (male, female), then randomized to one of the study arms using the randomizer with evenly presented elements in the Qualtrics^®^ XM platform.

### 4.2. Outcomes

The primary focus of this study was to assess the change in the PROMIS™ Sleep Disturbance 8A scale as the primary outcome (power calculation provided below). Additionally, the study evaluated the odds of achieving a minimal clinically important difference (MCID), defined as a reduction equal to or greater than half of the standard deviation of the baseline score. The MCID standard deviation criterion was calculated separately for each study arm.

As for secondary outcomes, the study examined changes in anxiety, stress, pain, and overall well-being. The secondary outcome assessment also included monitoring the number, type, severity, causality, and outcome of side effects, adverse events, and unanticipated problems for each study arm and for the overall study.

The change in sleep quality between the active and placebo product groups was evaluated using the PROMIS Sleep Disturbance 8A scale. Secondary outcomes were assessed using the following instruments: WHO 5 for overall well-being, PROMIS™ Anxiety 4A for anxiety levels, PEG for pain intensity and interference, and PROMIS Stress 4A for stress levels.

### 4.3. Safety

The assessment of spontaneously reported side effects in this study involved examining their frequency and severity. The severity of side effects was determined based on the utilization of medical services reported in response to the side effects. A grading schema was employed, following the Common Terminology Criteria for Adverse Events (CTCAE; v5.0 USDHHS): mild: no intervention (medication or medical advice) needed; moderate: a medication was taken due to the side effect or a participant sought medical advice from their HCP, urgent clinic or ED; severe: the side effect was medically significant but not life-threatening and/or the participant was admitted to the hospital for care and attention; life threatening: immediate medical intervention required and the participant was hospitalized, placed in the intensive care unit due to the side effect, and/or suffered long-lasting negative effects as a result of the side effect.

### 4.4. Covariates

Prior to conducting the analysis, three demographic variables (race, education, and ethnicity) were collapsed for simplicity. Race was recoded into the following categories: white, non-white (including participants identifying as Black, multi-racial, Asian, some other race, American Indian or Alaska Native, or Native Hawaiian or Pacific Islander), and prefer not to say. Education was recoded as either having a college degree (including participants with a bachelor’s or associate degree, and master’s or professional degree) or no college degree (including participants with less than high school, trade/technical/vocational degree, high school diploma without college, and some college without a degree). Ethnicity was recoded into the following categories: Hispanic, Non-Hispanic, and Prefer not to say. Baseline demographic variables, including age, recoded race, recoded ethnicity, recoded education level, sex assigned at birth (male, female), and body mass index (BMI; calculated from self-reported height and weight) were adjusted for in the analysis.

### 4.5. Power Analysis

To ensure adequate statistical power, a power analysis was performed using a general covariance structure and standard deviation to detect a meaningful difference in the change in our primary outcome in each study group relative to the placebo control. It was determined that a sample size of 198 participants in each study group would provide 85% power to detect a medium effect (Cohen’s d = 0.5) between each group relative to the placebo, with a two-sided *p*-value of 0.05 (corrected for multiple comparisons using Bonferroni). To account for conservative anticipated attrition levels (45%), recruiting up to 300 participants per study arm was planned to maintain an adequate sample size.

### 4.6. Statistical Analysis

A linear, mixed-effects regression model was used to assess the differences in the change in the variables of interest between each active product arm versus placebo. The parameter “na.action = na.omit” was set for each model, meaning that participants were excluded only from those models for which they did not have available data. All models were fit using an unstructured covariance matrix with a random-intercept at the participant level, and a random-slope at the study week level. The models tested the difference in the interaction between product arm and study week for active arm versus placebo, controlling for sex, age, race, ethnicity, and BMI. Post hoc Bonferroni-adjusted pairwise comparisons were used to assess the differences in the interaction between TruCBN™ formulation products and 4 mg of melatonin. Post hoc Bonferroni-adjusted pairwise comparisons were also used to assess the differences in the odds of achieving a MCID for sleep between each active product arm placebo, controlling for sex, age, race, ethnicity, education, and BMI.

### 4.7. Software

The Python programming language, version 3.95 (packages: pandas, version 1.4.3, and numpy, version 1.20.2) was used for data processing. R version 4.2.3 (packages: nlme, version 3.1-162, marginal effects, version 0.11.1, and tidyverse, version 2.0.0) was used to conduct the statistical analyses, and the package tableone version 0.13.2 was used to create Table 5.

## 5. Conclusions

The results demonstrate that CBN, consumed orally in a softgel with serving sizes of 25 mg, 50 mg, and 100 mg, could be a safe and effective alternative therapy for the improvement of sleep. There was no significant difference in improvements in sleep quality between any of the treatment groups and the 4 mg melatonin group, indicating that TruCBN™ offers an alternative for effective sleep support.

## Figures and Tables

**Table 1:** Participant sample summary at baseline.

	Softgel A	Placebo	Softgel C	Softgel B	Softgel D	t/χ^2^ *p*-Value
Variable	Mean (SD)/N (%)					
N	202	204	205	206	203	
Age	43.25 (13.61)	42.68 (12.00)	43.97 (12.70)	43.23 (12.93)	43.13 (12.59)	0.899
Race						0.387
White	174 (86.1)	160 (78.4)	167 (81.5)	165 (80.1)	153 (75.4)	
Non-White	26 (12.9)	42 (20.6)	36 (17.6)	38 (18.4)	47 (23.2)	
Prefer not to say	2 (1.0)	2 (1.0)	2 (1.0)	3 (1.5)	3 (1.5)	
Education: No College Degree	102 (50.5)	88 (43.1)	91 (44.4)	111 (53.9)	89 (43.8)	0.112
Sex At Birth: Male	93 (46.0)	93 (45.6)	95 (46.3)	94 (45.6)	93 (45.8)	1
Hispanic, LatinX, or Spanish origin						0.497
Non-Hispanic	183 (90.6)	186 (91.2)	182 (88.8)	185 (89.8)	183 (90.1)	
Hispanic	19 (9.4)	18 (8.8)	22 (10.7)	18 (8.7)	17 (8.4)	
Prefer not to say	0 (0.0)	0 (0.0)	1 (0.5)	3 (1.5)	3 (1.5)	
BMI	31.06 (8.72)	31.26 (9.65)	30.22 (8.25)	29.93 (7.04)	30.10 (7.26)	0.357
PROMIS Sleep Disturbance 8A	29.37 (6.16)	29.27 (6.08)	30.03 (5.68)	29.87 (6.52)	30.52 (6.46)	0.238
PROMIS Anxiety 4A	11.11 (3.38)	10.82 (3.46)	11.07 (3.44)	10.87 (3.47)	11.17 (3.36)	0.802
PROMIS Stress 4A	13.63 (3.62)	12.87 (3.83)	13.50 (3.46)	13.32 (3.81)	13.75 (3.49)	0.121
Pain, Enjoyment, General Activity Scale	5.80 (2.35)	5.51 (2.38)	5.37 (2.27)	5.59 (2.31)	5.17 (2.31)	0.383

**Table 2:** Significant factors associated with sleep disturbance: results from a linear mixed-effects regression model.

	Value	Std. Error	DF	t-Value	*p*-Value
(Intercept)	25.082	1.036	1857	24.213	<0.001
Education: No College Degree [Ref: College Degree]	1.499	0.364	1006	4.121	<0.001
Sex at Birth: Male [Ref: Female]	−0.666	0.361	1006	−1.847	0.065
Age	−0.007	0.014	1006	−0.468	0.640
Race: Non-white [Ref: white]	−0.573	0.472	1006	−1.213	0.226
Race: Prefer Not To Say [Ref: white]	0.160	1.634	1006	0.098	0.922
BMI	0.080	0.022	1006	3.679	<0.001
Hispanic, LatinX, or Spanish origin: Yes [Ref: No]	−0.794	0.640	1006	−1.242	0.215
Hispanic, LatinX, or Spanish origin: Prefer Not to Say [Ref: No]	−4.009	2.337	1006	−1.716	0.087
StudyWeek	−1.123	0.182	1857	−6.167	<0.001
Softgel A [Ref: Placebo]	−0.583	0.605	1006	−0.964	0.335
Softgel C [Ref: Placebo]	0.212	0.604	1006	0.351	0.726
Softgel B [Ref: Placebo]	0.113	0.601	1006	0.188	0.851
Softgel D [Ref: Placebo]	0.547	0.598	1006	0.914	0.361
Study Week: Softgel A	−0.564	0.258	1857	−2.188	0.029
Study Week: Softgel C	−0.603	0.254	1857	−2.371	0.018
Study Week: Softgel B	−0.544	0.250	1857	−2.176	0.030
Study Week: Softgel D	−0.566	0.249	1857	−2.275	0.023

The table presents the beta coefficients (β), standard errors (Std. Error), degrees of freedom (DF), t-values, and *p*-values for each variable. Higher values indicate a stronger positive association with sleep disturbance.

**Table 3:** Significant factors associated with anxiety: results from a linear mixed-effects regression model.

	Value	Std. Error	DF	t-Value	*p*-Value
(Intercept)	11.407	0.556	1190	20.502	<0.001
Education: No College Degree [Ref: College Degree]	1.141	0.197	1006	5.793	<0.001
Sex at Birth: Male [Ref: Female]	−0.277	0.196	1006	−1.414	0.158
Age	−0.051	0.008	1006	−6.561	<0.001
Race: Non-white [Ref: white]	0.088	0.255	1006	0.345	0.730
Race: Prefer Not To Say [Ref: white]	0.977	0.898	1006	1.087	0.277
BMI	0.032	0.012	1006	2.697	0.007
Hispanic, LatinX, or Spanish origin: Yes [Ref: No]	−0.116	0.346	1006	−0.336	0.737
Hispanic, LatinX, or Spanish origin: Prefer Not to Say [Ref: No]	−1.289	1.239	1006	−1.040	0.299
StudyWeek	−0.412	0.101	1190	−4.082	<0.001
Softgel A [Ref: Placebo]	0.101	0.315	1006	0.319	0.750
Softgel C [Ref: Placebo]	0.334	0.314	1006	1.066	0.287
Softgel B [Ref: Placebo]	−0.169	0.314	1006	−0.538	0.591
Softgel D [Ref: Placebo]	0.331	0.313	1006	1.057	0.291
Study Week: Softgel A	−0.095	0.138	1190	−0.691	0.490
Study Week: Softgel C	0.130	0.138	1190	0.943	0.346
Study Week: Softgel B	−0.028	0.136	1190	−0.205	0.838
Study Week: Softgel D	−0.232	0.135	1190	−1.720	0.086

The table presents the beta coefficients (β), standard errors (Std. Error), degrees of freedom (DF), t-values, and *p*-values for each variable. Higher values indicate a stronger positive association with anxiety, while lower values indicate a stronger negative association.

**Table 4:** Significant factors associated with stress: results from a linear mixed-effects regression model.

	Value	Std. Error	DF	t-Value	*p*-Value
(Intercept)	14.056	0.591	1256	23.797	<0.001
Education: No College Degree [Ref: College Degree]	0.974	0.209	1006	4.669	<0.001
Sex at Birth: Male [Ref: Female]	−0.715	0.207	1006	−3.448	0.001
Age	−0.069	0.008	1006	−8.416	<0.001
Race: Non-white [Ref: white]	−0.195	0.270	1006	−0.723	0.470
Race: Prefer Not To Say [Ref: white]	1.150	0.937	1006	1.228	0.220
BMI	0.050	0.013	1006	3.984	<0.001
Hispanic, LatinX, or Spanish origin: Yes [Ref: No]	−0.433	0.363	1006	−1.193	0.233
Hispanic, LatinX, or Spanish origin: Prefer Not to Say [Ref: No]	−1.906	1.285	1006	−1.483	0.138
StudyWeek	−0.353	0.095	1256	−3.699	<0.001
Softgel A [Ref: Placebo]	0.612	0.333	1006	1.840	0.066
Softgel C [Ref: Placebo]	0.637	0.331	1006	1.926	0.054
Softgel B [Ref: Placebo]	0.257	0.330	1006	0.777	0.437
Softgel D [Ref: Placebo]	0.776	0.330	1006	2.353	0.019
Study Week: Softgel A	−0.189	0.135	1256	−1.400	0.162
Study Week: Softgel C	−0.228	0.132	1256	−1.723	0.085
Study Week: Softgel B	−0.195	0.129	1256	−1.508	0.132
Study Week: Softgel D	−0.323	0.127	1256	−2.537	0.011

The table presents the beta coefficients (β), standard errors (Std. Error), degrees of freedom (DF), t-values, and *p*-values for each variable. Higher values indicate a stronger positive association with stress, while lower values indicate a stronger negative association.

**Table 5:** Validated measures for key outcomes used in Radicle Rest study.

Measure	Description	Scoring	How Was This Collected?
PROMIS Sleep Disturbance 8a	8-item measure assessing sleep disturbance (sleep quality) in the past 7 days	Scoring from 8 to 40, with higher scores translating to greater sleep disturbance	All participants received this measure within their weekly health surveys
PROMIS Anxiety 4a	4-item measure assessing frequency of anxiety symptoms in the past 7 days	Scoring from 4 to 20, with higher scores translating to greater anxiety	Participants who endorsed anxiety symptoms received this measure in their weekly health surveys.
PROMIS Stress 4a	4-item measure assessing frequency of stress symptoms in the past 7 days	Scoring from 4 to 20, with higher scores translating to greater stress	Participants who endorsed stress symptoms received this measure in their weekly health surveys.
PEG (Pain, Enjoyment, General Activity) scale	3-item measure assessing pain intensity and interference in the past 7 days	Scoring from 0 to 10, with higher scores translating to greater pain	Participants who endorsed pain symptoms received this measure in their weekly health surveys.

## Data Availability

The datasets used and/or analyzed during the current study are not publicly available due to privacy restrictions; however, please contact the corresponding author if you are interested and we will consider reasonable requests.
